# Acute home-based care for patients with cancer to avoid, substitute, and follow emergency department visits: a conceptual framework using Porter’s Five Forces

**DOI:** 10.1186/s44201-022-00008-3

**Published:** 2022-07-01

**Authors:** Christopher W. Baugh, Stephen C. Dorner, David M. Levine, Nathan R. Handley, Kathi H. Mooney

**Affiliations:** 1 Department of Emergency Medicine, Brigham & Women’s Hospital, Neville House 2Nd Floor, 75 Francis Street, Boston, MA 02115 USA; 2 Department of Emergency Medicine, Massachusetts General Hospital, Boston, MA USA; 3 Department of Medicine, Brigham & Women’s Hospital, Boston, MA USA; 4 Department of Oncology, Thomas Jefferson University, Philadelphia, PA USA; 5 Huntsman Cancer Institute, University of Utah, Salt Lake City, UT USA

**Keywords:** Oncology, Cancer, Emergency department, Home-based care, Home hospital, Telemedicine, Crowding

## Abstract

**Background:**

Patients with cancer constitute a large and increasing segment of patients who receive unscheduled hospital-based care due to treatment-related symptoms and disease progression. The initial hospital-based touchpoint for these unscheduled hospitalizations is often the emergency department. Traditional models of emergency department and inpatient hospital-based care are saturated and incapable of scaling to accommodate the future, increased needs projected for this population. New models of care are necessary to address this gap. Acute home-based care is a promising tool potentially providing patient-centric, efficient care to eligible patients.

**Methods:**

We applied Porter’s Five Forces framework that addresses the bargaining power of buyers and suppliers, threat of substitutes and new entrants, and industry rivalries plus the sixth force of regulation to clarify the factors that will promote or challenge the adoption of a home-based cancer care referral model before or following emergency department visits. Exploring this framework provides insights into the complexities of scaling an acute home-based cancer care model and highlights ways for health systems including hospitals, emergency departments, physician groups, and individual emergency physicians and oncologists to optimize their roles in this emerging model of care.

**Results:**

We found that current workforce shortages, as well as workflow, infrastructure, and regulatory complexities, pose major challenges that unless carefully addressed may restrict the growth of acute home-based cancer care. Additional uncertainties persist around appropriate payment models and the competitive landscape. Key promoting factors include the recognized need in the cancer community and among payers for new models to decrease unscheduled hospitalizations and emergency department visits as well as the uptake of home-based and technology-enabled solutions during the COVID-19 pandemic. A better understanding of these forces helps to clarify the risks and opportunities as new entrants build their programs.

**Conclusions:**

Acute home-based cancer care is a promising tool to complement traditional outpatient clinics, emergency departments, and inpatient hospital-based models of cancer care. New technologies and policies increasingly enable a broader scope of cancer care in the home setting.

## Background

Over the course of the twentieth century, the site of medical treatment transitioned away from individuals’ homes and into centralized centers for medical care. Since the 1950s, as diagnostic and treatment technologies advanced, doctor’s tools no longer fit into a neatly packed black bag but, rather, are concentrated in health centers, clinics, and hospitals [1]. Correspondingly, emergency departments (EDs) arose as the hospital-based point of entry for rapid access to diagnostics and treatments. Unlike most clinics, EDs provide around-the-clock access to clinicians whenever the need may arise. Today, there are over 5000 EDs in the United States (US), roughly 1100 of which are associated with academic teaching hospitals and 1300 of which provide critical access to essential medical care in areas where there otherwise would be none [2, 3]. However, the traditional ED-to-hospital pathway has in some cases exacerbated congestion and cost in US health care, leading some systems and communities to decentralize and distribute care to patients’ homes [4]. Examples of this phenomenon include the adoption of telehealth clinic visits, home hospital programs, and increased access to home infusion services [5, 6]. Payers are also structuring incentives for both patients and hospitals to drive care to lower cost settings. In addition, with the COVID-19 pandemic, the Centers for Medicare and Medicaid Services (CMS) developed the Acute Hospital Care at Home Waiver temporarily allowing hospital-level Medicare reimbursement for the same care at home [7]. As a result, we are increasingly witnessing the site of care transition back to the home.

**Fig. 1:**
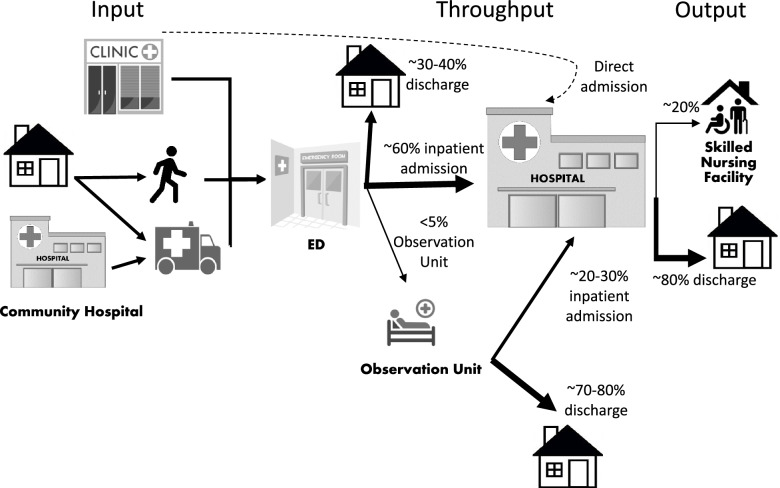
Traditional hospital-based care model

Currently, in the US, about 4.2% of all ED visits are made by a patient with cancer [8]. In 2017, there were an estimated 15.5 million patients with cancer diagnoses and that population is anticipated to grow to 25–30 million in 2040 [9]. That burgeoning patient population also requires frequent, high acuity medical care due to the impact of disease progression and treatment side effects including fatigue, pain, fever, dehydration, dyspnea, and infection-related syndromes, among others [10]. Patients with cancer have a rate of traditional hospital admission roughly quadruple that of patients without cancer (~ 60% vs 16%) [8]. In Fig. 1, we illustrate the traditional input, throughput, and output flow of patients with cancer requiring unscheduled hospital-based acute care via the ED. The growing size of the population of patients with cancer and their high rate of unplanned health care utilization suggests that targeted approaches to care to avoid, substitute, or follow ED care would be beneficial. Home-based care is one such approach with three main use cases: the potential avoidance of an ED visit via a telehealth evaluation; the replacement of an ED visit via in-home diagnostics and treatments, such as those coordinated by community paramedics; and an alternative to hospitalization through admission to a hospital at home program.

Patients at high risk for accessing acute unscheduled care could be proactively engaged with home-based care models to reduce the incidence of ED visits and resulting rehospitalizations. Predictors associated with urgent care and emergency department use, such as treatment (e.g., recent chemotherapy), cancer type, and previous pattern of acute unscheduled care utilization could help target the optimal patients for the care program [11]. Risk prediction models translated into effective tools leveraged at multiple points prior to, during, and after an acute unscheduled care visit are needed [12]. Telehealth visits involving remote synchronous or asynchronous communication between patient and outpatient clinicians delivering longitudinal care are not new, historically consisting primarily of telephone calls to discuss new symptoms or follow up on a recent visit. As new technologies have become more ubiquitous, additional methods of remote communication have allowed for additional encounters, such as electronic mail, patient gateway messaging, and video teleconference visits to supplement patient calls. While these visits do not bring diagnostic testing or therapeutics into the home, clinicians can order urgent outpatient testing, write prescriptions, and address a wide variety of straightforward clinical scenarios using these tools to help prevent avoidable ED visits.

Furthermore, increasing sophistication of in-home diagnostics (e.g., point of care ultrasound and laboratory studies, X-rays) and a wider array of therapeutic options (e.g., intravenous medications and infusions) are making more patients with complex needs eligible for episodic paramedic-enabled home-based visits or active acute treatment through home hospital care that could replace some ED visits and hospitalizations for patients with cancer [13, 14]. This type of home-based care is best accomplished after an eligibility screen based on the clinical scenario, since only a limited subset of ED therapeutics and diagnostics are currently available in the home. For example, home-based computed tomography (CT) scans are out of scope but are a common diagnostic test ordered in the ED for patients with cancer for typical complaints such as abdominal pain, headache, and chest pain.

As a substitute for traditional hospitalization, the provision of hospital-level care at home has been explored as a care delivery model in the US since the early 1990s at Johns Hopkins University, Veterans Affairs Hospitals and various other hospitals around the country [15]. Over the last 20 years, an increasing number of investigators have explored the safety, efficacy, perception, and efficiencies that are afforded by hospital care at home. Researchers have identified shortened length of stay, decreased costs, greater patient satisfaction, and higher rates of physical activity with hospitalization at home compared with traditional hospitalization [16–22]. Furthermore, emergency physicians are willing to embrace alternatives to traditional hospital-based care when provided with the programmatic support to ensure continuity of care through a safe transition plan [23, 24].

Historically, home hospital programs have excluded patients with cancer. In fact, there are few models of any type of home services for patients with cancer except for the well-established home hospice and palliative care model. Recently though, there has been growing interest in the home as a site of cancer care. In 2020, Penn Medicine described their Cancer Care at Home program that effectively provides a home infusion of a variety of cancer drugs [25]. With the increasing focus on treatment models that reduce the need for unscheduled cancer care, commentaries also began to advance the potential role of hospital at home programs [26, 27]. Huntsman Cancer Institute’s Huntsman at Home program began in 2018 as the first cancer-specific hospital at home program. A recent program description and outcome evaluation demonstrated the appropriateness of home hospital models for the cancer population and significant decreases in subsequent ED use and rehospitalization along with cost savings [28]. The Huntsman model addresses the need for both short-term (3–7 day) acute hospital-level treatment at home and 30-day subacute care in order to prevent exacerbations that often occur with cancer symptom fluctuations and disease progression, resulting in rebound ED use [29]. Because these exacerbations often have a rapid and unpredictable onset, ongoing monitoring and rapid response teams are important features for at-home cancer care programs.

Home-based care related to acute unscheduled medical needs of patients with cancer is an evolving care delivery option. However, many challenges remain for this delivery innovation to continue to scale and extend to all eligible patients. Shifting the site of care to the home in these cases is disruptive for numerous stakeholders including patients, caregivers, clinicians, health systems, payers, and regulators. The incentives and interactions of these groups are complex. As a result, we need strategic tools to identify and understand these interdependencies. One of the most useful ways to account for complexity is to apply a framework to systematically examine these relationships. In this analysis, we explore home-based care for patients with cancer with a focus on unscheduled acute-level cancer care needed before, after, or as a substitute for an ED encounter through the lens of one of the best known frameworks for this purpose, Porter’s Five Forces.

## Methods

**Fig. 2:**
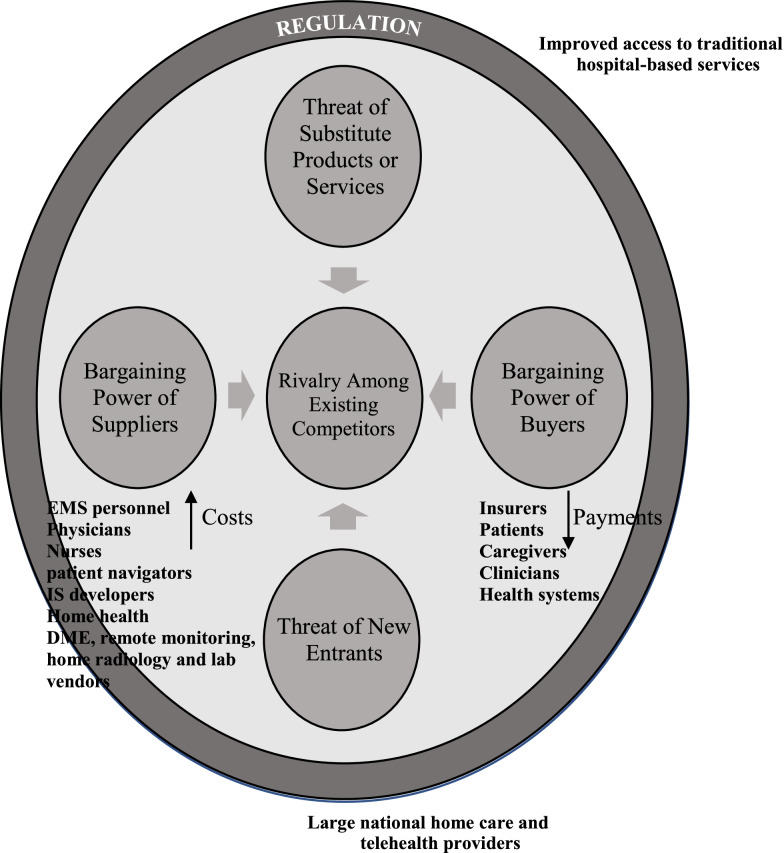
Porter’s Five Forces

We convened an interdisciplinary group of authors with direct experience in financing, launching, and sustaining acute home-based care programs, business training, and work backgrounds (e.g., management consulting, Masters in Business Administration degrees), and clinical backgrounds in oncology, medicine, and emergency medicine to iteratively apply Porter’s Five Forces to home-based acute level care for patients with cancer. All authors contributed to serial revisions and communicated via a variety of virtual platforms such as electronic mail and video conferencing to reach consensus. First described by Michael Porter in a 1979 Harvard Business Review article and illustrated in Fig. 2, Porter’s framework continues to shape business practices and academic thinking today [30]. A Five Forces analysis can help organizations assess industry attractiveness, how trends affect industry competition, and strategies to best compete and position themselves to succeed. For many industries, including health care, regulation is a relevant sixth force to consider. Though industries vary, the underlying drivers of sustained growth and financial viability are constant.

While profitability is not necessarily a goal of hospital-administered home-based care initiatives, financial sustainability to ensure that program costs are matched by appropriate payments is critical to ensure the uptake and longevity of these programs. Until recently, regulatory and fee-for-service payment models have not accounted for the shift in site-of-care to the home, particularly for payments covering acute and subacute episodic care. As a result, until CMS authorized the temporary Hospital-at-Home Waiver, hospital subsidies were needed to finance these programs. Where such programs create indirect value for hospitals, such as easing bed capacity challenges in traditional settings, hospitals may be willing to subsidize programs to some extent; however, programs dependent on subsidies are perpetually vulnerable, often struggle to scale, and are not fairly reimbursed for the value they bring to the healthcare system.

## Results

### Supplier power

This force requires us to identify suppliers in the home-based acute care space for patients with cancer and assess their ability to raise costs. Supplier control of prices is a function of the number of suppliers for each essential input, the uniqueness of their product or service, their relative size and strength, and the cost of switching between suppliers.

Suppliers in this service line include the workforce of emergency medical services (EMS) personnel, physicians, nurses, patient navigators, and information services (IS) vendors and developers, and also those who have current expertise in home services, including home health agencies and those supplying durable home medical equipment (DME), remote monitoring technology, home radiology and labs, and home courier services. In addition, staffing costs and availability play a critical role in the scalability of acute home-based care, for example, current registered nurse shortages, especially nurses with acute care experience interested in providing home-based care. Other challenges may include staffing costs with the need for payment parity with traditional care delivery models.

The attractiveness of home-based models of care as a worksite for clinicians compared to the traditional clinic and hospital setting is yet to be determined. Some clinicians will prefer the comradery and immediate backup of the traditional hospital setting while others will prefer the independence and satisfaction of working with patients in their home environment. In addition, for home programs that include a significant component of virtual care delivery, the attractiveness of remote work (e.g., lack of commute, increased family time) combined with high burnout rates among clinicians in traditional settings may make telehealth delivery an attractive alternative career path. The training and experience of staff delivering care in a home-based cancer care model also require attention; transitional home-hospital programs that have not included patients with cancer are typically staffed by internal medicine trained physicians to mirror a hospitalist model. On the other hand, urgent care and mobile response program telehealth visits aimed at episodic care to avoid ED visits or to affirm the safety of an ED discharge plan are typically staffed by emergency physicians. The oncology home-based model will evolve with a combination of medical expertise and warrants further study. The generalist model with hospitalists and emergency physicians can be augmented by oncologists who understand issues of disease progression and their treatment implications. In addition, optimal delivery models are yet to be determined and may include care delivery by advanced practice providers (APP) with physicians providing backup expertise. In any form, interaction with the patient’s oncology team is indicated and underscores the need for continuity and communication when integrating a new site of care such as the home.

As a new site of care, new workflows and infrastructure will be required to enable acute home-based care. For example, a clear process to enable appropriate and efficient ED transfers to acute home-based cancer programs is an essential component. Furthermore, clinicians need IS developers to integrate home-care tools such as documentation, video conferencing, and billing and coding enhancements into the existing electronic medical record (EMR) platforms to enable remote care. Current hospital EMR systems lack effective integration with home-based settings. The current labor shortage, supply chain inefficiencies, and need for home site integration into health systems enhance supplier power and without proper recognition and planning represent a significant challenge to scaling home-based cancer programs.

### Buyer power

This force requires us to identify buyers in the home-based acute care space for patients with cancer and assess their ability to lower costs (or payments). Buyer control over payments is driven by the number of buyers in the market, the relative value of each individual buyer to the organization, and the cost to the buyer of switching between suppliers. In markets with fewer influential buyers, those buyers are often able to dictate terms. The primary buyers of home-based cancer care are payers. As described previously, acute home-based cancer programs have not been widely integrated into US health care, primarily because reimbursement models have not adequately compensated for intensive home-based care that would otherwise require hospitalization. Additionally, government provisions at the local, state, and federal levels associated with the COVID-19 public health emergency (including payment parity between telehealth visits and in-person visits and the CMS Waiver for Hospital at Home payment at bundled hospital inpatient levels) were largely temporary and will terminate when the pandemic health emergency is withdrawn. Further CMS or legislative action will be needed to extend payments and regulations beyond the pandemic period.

In 2015 the American Society of Clinical Oncology (ASCO) released the Patient Centered Oncology Payment (PCOP) model, which featured bundled payments and a focus on four key areas: 1. Avoidance of ED visits and inpatient hospitalizations, 2. Adherence to evidence-based appropriate use criteria, 3. Patient-directed end of life care and 4. Quality of care performance [31]. Expanding acute home-based care was not an explicit component of the PCOP model, but a cost-effective and equitable delivery model would complement its main goals. Indeed, the PCOP model calls for team-based care with navigation resources, and the innovative role of digital health navigators would be a natural extension of the traditional case manager role [32]. However, when virtual care is augmented by in-home assessment, diagnostics, and treatments, the substitutive capabilities of home-based virtual care are transformed to more closely match traditional clinic-based in-person care. Similarly, cancer home hospital programs offer a safe equivalent to the more expensive traditional hospitalization.

Patients, caregivers, clinicians, and health systems can also be considered buyers in this space. Their perceptions of the capabilities of home-based care options need to be addressed, since as an entirely new approach, many may hold onto views that hospital-based care is a safer option [33, 34].

### Competitive rivalry

This force requires us to identify the number and capability of competitors in the market for home-based cancer acute care. The biggest competitor for acute home-based care are the status quo of ED visits and hospitalization. Home-based care is disruptive to the traditional pathway of hospitalization after ED assessment and will pull patients away from the inpatient setting. This may be welcomed by stakeholders with inpatient demand exceeding capacity but opposed by stakeholders with excess inpatient capacity or those planning future facility growth (e.g., a hospital that has funded a large expansion of on-campus inpatient beds) as a threat to their business model. With new home-based cancer care models emerging in the market, competition will offer both general home hospital models and cancer-specific opportunities. New home-based models of care have also caught the interest of venture capital and health care start-up businesses. Currently, there are several general home hospital vendors and at least one that is cancer-specific. These models may be of interest to health systems who do not have home-based expertise to stand up a program themselves and therefore prefer to turn to an external partner. While this may accelerate adoption and rapid scaling, it will create a competitive rivalry.

### Threat of substitution

This force requires us to identify close substitutes in the market for home-based cancer acute care. Access to close substitutes makes customers more price-sensitive. Substitutes reduce both the power of suppliers and the attractiveness of the market. In a telehealth visit that is limited to provider-patient communication only, there is a narrower range of substitutive use cases, for example, discussion of lab or imaging results or symptom management recommendations. Among patients in need of palliative or hospice care, substitutes for hospital-based acute care or inpatient hospice may be less aligned with patient and family preferences for home-based care. Previous research suggests that about a fifth to a quarter of ED visits among patients receiving palliative care for terminal cancer were potentially avoidable and could have been substituted with alternative encounters [35–37]. Patients receiving end-of-life home-based care acquired several benefits including slightly increased patient satisfaction at a 1-month follow-up, slightly reduced health care cost, fewer visits to outpatient clinics, a small reduction in inpatient days, and an increased likelihood of dying at home [38]. Home palliative care of terminally ill patients seeking care for the symptomatic management of dyspnea is another use case to safely redirect care while also aligning with patient goals of care [39]. Direct enrollment in palliative care programs during an ED visit is another feasible strategy that has been associated with increased quality of life without shortening survival, suggesting that extending this strategy to a direct to home-based palliative care program may be feasible [40]. Substitution can be addressed through a comparative study of fit and quality. It is also incumbent that cancer home hospital programs make a clear delineation of their service model and targeted cancer populations.

### Threat of new entry

This force requires us to identify the threat of new entrants into the market for home-based acute care space for patients with cancer. New entrants threaten the profitability of established incumbents. Resilient barriers to entry, such as patents or economies of scale, protect incumbents. As more home-based care options enter the market, patients and their oncology clinicians may increasingly have multiple options that promise to deliver similar monitoring, diagnostic, and treatment capabilities. Home-based care programs affiliated with outpatient oncology practices will be a default option, especially due to EMR interoperability advantages as EMRs consolidate; however, emerging third-party services may step in to compete as previously described. Over time, if regulatory, technology, and staffing barriers are lowered, competitive services that extend to larger networks could come to market and drive down both costs and payments. For example, a large telehealth provider could enter the home-based cancer care business and leverage their experience and technologies to complete with local programs at a lower cost. As a result, smaller organizations may choose to partner to outsource home-based services rather than internally develop their own programs.

#### Regulation

Regulation is a sixth force beyond the core Five Forces relevant for many industries, including the home-based acute care space for patients with cancer. Regulatory restrictions may dictate the acceptability of telehealth communication platforms, control administration of scheduled substances in the home setting, impose medical licensure requirements that complicate inter-state delivery of virtual care, and create an administrative burden for virtual health and in-home programs. In addition, there are many restrictive regulations that impact care in the home, such as home-bound status to receive services, and conversely, there are many hospital-based regulations, for example, the requirement of nurses on the premises 24/7, that do not transfer when acute level care is provided at home. Differences among state-specific regulations also present a barrier to scale to multiple states. To successfully establish the home as a legitimate site for cancer care moving forward, both state and federal laws and regulations will require revision. Finally, medical malpractice coverage typically extends to home-based care, obviating the need for clinicians to seek new policies or amend existing ones as they begin to provide care in this new model. Considerations such as creating accurate and timely documentation of encounters, maintaining practice within the scope of care supported by roles and educational background, and supporting a quality improvement program to review cases are critical elements for both traditional and home-based care service lines.

## Discussion

**Fig. 3:**
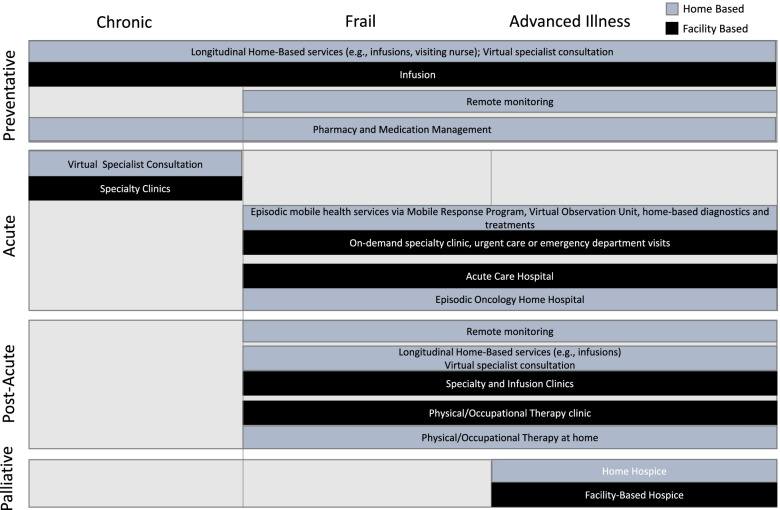
Overlap of home-based oncology care with traditional inpatient and outpatient care

The population of patients with cancer seeking acute unscheduled care is rapidly expanding. Simultaneously, the current crowding of EDs and traditional hospitals will likely persist, making traditional care models unlikely to meet patients’ needs. Expanding the availability and capabilities of home-based cancer care is a promising new approach that offers the potential to bridge the growing gap between the demand and supply of acute cancer care [28]. In Fig. 3, we illustrate the expanding spectrum of home-based care with a focus on unplanned acute cancer care related to ED visits.

Our analysis reveals several important findings about home-based care for patients with cancer. First, our workforce is a key supplier that needs to adapt to scale both remote monitoring and in-home care capabilities. Re-alignment of incentives and strategic support of training programs will likely be necessary to build this future workforce. Next, current payment models that inadequately compensate for home-based delivery or are explicitly temporary or limit applicability to patients based on an insurance carrier create a risk for hospitals to invest in building home-based care programs. In addition, uncertainty remains around whether consolidation of home-based care programs with an outsource model versus a distributed model with a network of individual programs associated with traditional hospitals will emerge. As a result, many hospitals may continue to take a “wait and see” approach for the foreseeable future. Finally, the future of home-based care is particularly dependent on legislative and regulatory actions that may enable, or alternatively, impose barriers to home-based care.

Home-based care has several advantages and disadvantages with respect to patients far away from hospitals. The use case for geographically distant care should be a priority for study. It is important that new models of cancer delivery, such as home-based care, not perpetuate access inequities in their design. Virtual care can extend to anywhere with an internet connection; however, for some low-income rural communities, internet access is not a given, which exacerbates health disparities unless addressed as part of the care model. As a substitute for emergency care and inpatient hospitalization, access to in-home health care providers, diagnostics, and treatments is typically required, thus home-based services at a distance from cancer facilities likely need a combination of virtual and on-ground resources. Response times and transport times are relevant to operate an efficient and safe service. In addition, some vulnerable populations such as the undomiciled, patients with fewer social supports at home, and those with new or exacerbated disabilities that require continuous nursing care may be better served by traditional hospital services, unless care support is provided in the model (as it is in hospital care design) and reimbursed accordingly. Notably, home hospital and mobile response programs typically leverage cellular data networks and lend patients hardware to bridge disparities in access to these technologies. Furthermore, family members who live with patients may be able to bridge the gaps in technology adoption due to disparities in health literacy, tech literacy, etc. All of these factors require consideration as rural models are under development [41, 42]. Huntsman at Home’s cancer-specific model was recently adapted for rural delivery in three distant communities that are a 2- to 5-h, one-way drive from Huntsman Cancer Institute and involve both telehealth and on-ground components. In addition to adapting models to address access inequities, home-based programs, whether rural or in low-income communities, allow clinicians to see the context in which patients are dealing with cancer and its treatment demands and, therefore, have the potential to better identify and address social determinates of health and overcome some of the difficulties from the power differential that occurs when patients leave their homes and communities to receive care on the “turf” of clinicians.

Looking ahead, we should leverage currently operating home hospital, mobile response, and telehealth programs to evaluate capabilities to include patients with cancer and encourage further development of cancer-specific home care models. Further work is urgently needed to develop and test alternate payment models that appropriately reimburse the full spectrum of home-based care. Support is also needed to incentivize health system infrastructure improvements and workflow changes to rapidly integrate the home as a site of care, squarely in the mainstream of health care. Furthermore, the COVID-19 public health emergency accelerated home hospital and telehealth adoption and this momentum should not be lost. Further research is needed to better identify conditions and patients who would most benefit from alternatives to short inpatient hospitalization. New models should also be studied in a variety of settings (e.g., urban and rural, academic and community, safety net, critical access) to better understand the generalizability of cancer home-based care to all patients who would benefit from it. With an eye toward the end of the national COVID-19 public health emergency, CMS, Congress, and the hundreds of hospitals across the country who have begun hospitalizing patients at home must determine whether home-based hospitalization has a future role in the healthcare landscape. In the context of progressive demographic and social transitions toward older ages and a higher prevalence of chronic diseases, the COVID-19 pandemic has exacerbated long-known problems of hospital-centered health systems lacking strong home-based care services.

## Limitations

While the Five Forces framework is typically applied in business settings outside of healthcare, it does offer relevant insights for the home-based cancer care service line example. Furthermore, as an evolving service line with dynamic laws, policies, and technologies governing the requirements and abilities of home-based cancer care models to meet patients’ needs, the framework we discussed in this analysis will certainly require updating in the future to remain relevant and accurate. Perhaps the most significant unknown element is the future landscape of regulation and reimbursement for telehealth and home hospital services, since more permanent and clear funding support will be needed for new entrants and the full potential of these programs to be realized. Finally, we did not leverage a systematic process, such as the Delphi method, to reach a consensus. However, given the relatively small size of the author group, we found our method to be an efficient and equitable way for all authors to iteratively contribute to the final analysis.

## Conclusions

Patients with cancer face fluctuating treatment and disease symptoms that result in unplanned acute care episodes. Home-based care programs can safely address many of these needs, preventing ED use and rehospitalization, and at a lower cost. The Porter’s Five Forces framework helps to identify potentially challenging market forces at play in the home-based care space for cancer care. This is expected from a disruptive health care innovation that requires the inclusion of a new site-of-care and necessitates changes to workflows, infrastructure, policy, and payment models. Recognition of and attention to these forces is critical to scale and sustain home-based cancer care. As the dramatic consequences of the COVID-19 pandemic have drawn attention to the flaws of our traditional health systems while also stimulating new home-based care programs, this is the moment to maintain momentum and implement more permanent policy changes and infrastructure to support innovative cancer care delivery models.

## Data Availability

N/A.
